# Postoperative Pyoderma Gangrenosum Mimicking Early Periprosthetic Joint Infection After Total Hip Arthroplasty in a Patient With Hairy Cell Leukemia: A Diagnostic Challenge

**DOI:** 10.7759/cureus.111606

**Published:** 2026-06-27

**Authors:** Richard Lehnert, Stephanie Schneider, Bernhard Walker, Daniel Schrednitzki

**Affiliations:** 1 Orthopedics, Traumatology, Hand and Reconstructive Surgery, Sana Hospital Lichtenberg, Berlin, DEU; 2 Orthopedics, Traumatology, Hand and Reconstructive Surgery, Sana Hospital Berlin, Berlin, DEU; 3 Orthopedics,Traumatology, Hand and Reconstructive Surgery, Sana Hospital Lichtenberg, Berlin, DEU

**Keywords:** hematologic malignancy, periprosthetic joint infection, postoperative complication, pyoderma gangrenosum, total hip arthroplasty

## Abstract

Pyoderma gangrenosum (PG) is a rare, sterile neutrophilic dermatosis characterized by rapidly progressive, painful cutaneous ulcerations and frequently associated with systemic diseases, including inflammatory bowel disease, rheumatologic disorders, and hematologic malignancies. In particular, recognition of PG as a cutaneous manifestation of an underlying hematologic neoplasm can be critical for timely diagnosis of the systemic disease. Postoperative PG after total hip arthroplasty may closely mimic early periprosthetic joint infection and can lead to unnecessary surgical revisions and prolonged antibiotic therapy.

An 83-year-old woman underwent elective right total hip arthroplasty for end-stage osteoarthritis. Her history included arterial hypertension and mild leukocytopenia; the left hip arthroplasty 10 years earlier had been uncomplicated. The index procedure and early postoperative course were initially uneventful. On postoperative day 6, erythema developed around the wound, followed by pustule formation, purulent-hemorrhagic secretion, and a secondary rise in C-reactive protein (CRP). Early periprosthetic joint infection was suspected, and the patient underwent two revision surgeries with extensive debridement, head and liner exchange, and broad-spectrum antibiotics. Intraoperatively, inflammation remained confined to skin and subcutaneous tissue without fascial or muscular involvement, and microbiological cultures were largely negative or yielded organisms interpreted as contaminants. Dermatologic consultation and histopathologic examination ultimately confirmed PG. Negative-pressure wound therapy was discontinued, topical wound management was continued, and systemic corticosteroids were initiated after early granulation tissue formation, resulting in gradual local improvement and secondary wound healing. Given the known association between PG and hematologic malignancies, a targeted hematologic work-up was performed and subsequently revealed an underlying hairy cell leukemia, providing a unifying explanation for the patient’s chronic leukocytopenia. Postoperative PG after elective total hip arthroplasty is a diagnostic challenge that may be misinterpreted as early periprosthetic joint infection. Atypical wound deterioration with severe pain, superficial distribution of inflammation, negative cultures, and pathergy after surgical interventions should raise suspicion of PG and prompt dermatologic and histopathologic evaluation. In addition, clinicians should be aware of the strong association between PG and hematologic malignancies and pursue appropriate hematologic assessment when clinically indicated. Early recognition and timely immunosuppressive treatment are crucial to avoid repeated surgical trauma and to facilitate prompt identification and management of associated systemic diseases such as hairy cell leukemia.

## Introduction

Pyoderma gangrenosum (PG) is a rare neutrophilic dermatosis characterized by painful, necrotic skin ulcerations and classified as an orphan disease ​[1, 2]. Epidemiologic data from a prospective Italian multicenter study reported an incidence of approximately 5.2 new cases per million inhabitants per year, confirming that PG is rare but likely underdiagnosed ​[1]​. The median age at presentation is 59 years, with a female predominance reported in up to 76% of cases ​[2]​. 

Clinically, classic PG often begins as sterile, painful pustules that may arise after minor trauma or surgical procedures (pathergy phenomenon) and quickly progress to ulcers with purulent or hemorrhagic exudate and intense pain ​[1, 3]. PG is frequently associated with systemic disorders such as inflammatory bowel disease, rheumatoid arthritis, and hematologic malignancies, including myelodysplastic syndrome, leukemia, and lymphoid neoplasms ​[2, 4]. Recent data suggest that patients with hematologic malignancy-associated PG have a particularly unfavorable overall prognosis compared with those without malignant disease ​[4]. 

Post-surgical PG is an uncommon but increasingly recognized entity in which PG develops at or near a surgical site and closely mimics wound infection or necrotizing soft-tissue infection ​[3, 5, 6]. In a systematic review of 31 cases after orthopedic or traumatologic procedures, most lesions involved the lower limb, symptom onset ranged from two to 17 days postoperatively, and initial misdiagnosis as surgical site infection was the rule rather than the exception ​[5]. Several case reports have documented PG following hip arthroplasty or hemiarthroplasty, where repeated debridements and component exchanges were performed for presumed periprosthetic joint infection before PG was recognized ​[6-8]. 

We present a case of postsurgical PG following elective total hip arthroplasty to highlight the importance of recognizing this rare but critical differential diagnosis and to emphasize clinical features that may help distinguish it from periprosthetic joint infection. 

## Case presentation

Patient information 

**Figure 1:**
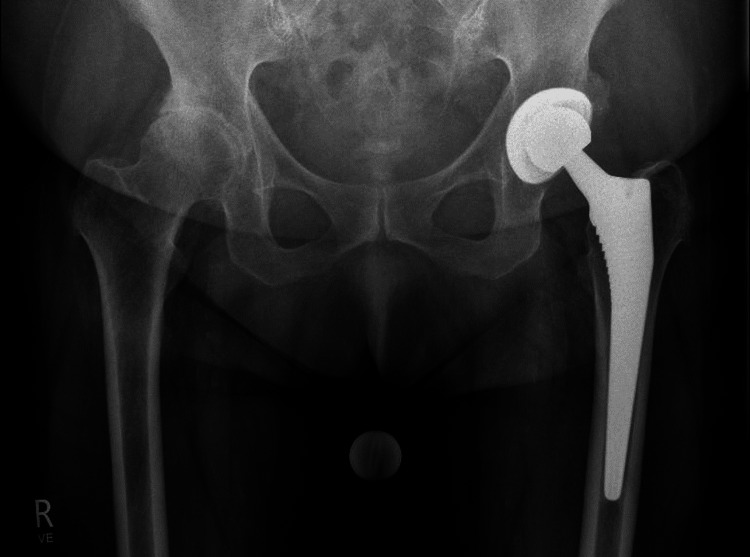
Preoperative anteroposterior radiograph of the pelvis showing advanced osteoarthritis of the right hip with joint space narrowing, subchondral sclerosis, and osteophyte formation. The left total hip arthroplasty, performed 10 years earlier, appears well-fixed with no signs of loosening.

An 83-year-old woman presented with a three-year history of progressively increasing pain and restricted range of motion in the right hip. Radiographs demonstrated advanced osteoarthritis of the right hip (Figure 1). Her left hip had been replaced with a total hip arthroplasty 10 years earlier without any complications. Relevant medical history included arterial hypertension; there was no known inflammatory bowel disease, connective tissue disease, or previous dermatologic disorder. Preoperative laboratory testing revealed a mild leukocytopenia, while other parameters were within normal limits. Preoperatively, the patient had a white blood cell count of 3.9 × 10⁹/L with platelets of 101 × 10⁹/L and hemoglobin 6.6 mmol/L. During the postoperative course, the white blood cell count remained around 3.6-3.8 × 10⁹/L, with relative lymphocytosis and stable hemoglobin and platelet levels.

Surgical procedure and early postoperative course 

**Figure 2:**
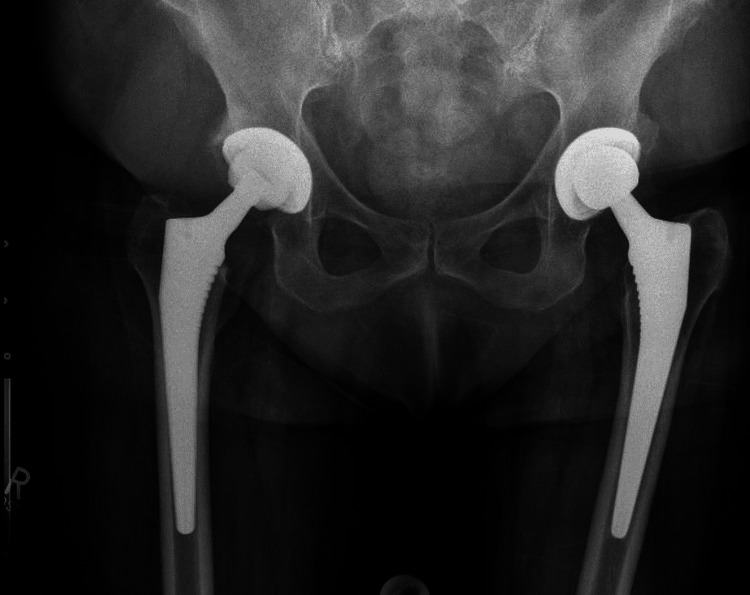
Postoperative anteroposterior radiograph demonstrating satisfactory component positioning and alignment after implantation of the right total hip arthroplasty.

Elective right total hip arthroplasty was performed through a standard approach (Figure 2). Implantation of the components and wound closure proceeded without intraoperative complications. In the immediate postoperative period, pain management, mobilization, and the wound appearance were unremarkable, and the patient recovered as expected. Postoperative pain was initially mild and consistent with the expected course after total hip arthroplasty.

On postoperative day 6, discrete erythema appeared around the wound margin. At that time, the C-reactive protein (CRP) level had decreased from 183 mg/L on postoperative day 2 to 128 mg/L. On postoperative day 7, however, the erythema increased, and pustule formation with purulent-hemorrhagic secretion from the wound was observed. Notably, the patient developed severe burning incisional pain that was disproportionate to the superficial clinical findings, raising concern for an underlying pathological process. Concomitantly, the CRP level rose again to 160 mg/L. Table 1 summarizes the temporal relationship between CRP levels, key clinical events, and therapeutic interventions.

**Table 1:** Temporal relationship between postoperative clinical events, investigations, and C-reactive protein (CRP) levels

Postoperative day	Clinical event/intervention	CRP (mg/L)
0	Elective right total hip arthroplasty (THA); uneventful immediate postoperative course	–
2	Peak postoperative CRP after primary THA	183
6	Onset of discrete erythema around the wound; pain was still consistent with the expected postoperative course	128
7	Increase in erythema, pustule formation, purulent-hemorrhagic secretion; severe, disproportionate wound pain; suspicion of early periprosthetic joint infection	160
8	First revision (extensive debridement, head and liner exchange); initiation of intravenous cefuroxime	–
10	Persistent wound deterioration; second revision; multiple cultures and histopathology obtained; CRP peak under ongoing suspicion of infection	223
13	Application of negative-pressure wound therapy (vacuum-assisted closure (VAC)); switch to intravenous ampicillin/sulbactam	–
18	Dermatology consultation; biopsy confirming pyoderma gangrenosum; discontinuation of VAC; continuation of topical wound management	151
21	Initiation of systemic corticosteroid therapy after early granulation tissue formation	72
54	Advanced wound healing with secondary closure; CRP near normal	4

First revision surgery 

Given the clinical picture of wound deterioration and rising inflammatory markers, an early periprosthetic joint infection was suspected. On postoperative day 8, the patient was taken to the operating room for revision. The previous incision was excised, and distally lividly discolored skin was noted along the wound. No typical seroma cavity was identified in the subcutaneous tissue, but smeary necrotic areas were present. Extensive debridement of nonviable tissue was performed, and the wound was irrigated with jet-lavage. 

The fascial and muscular layers appeared macroscopically intact and closed, with no obvious signs of deep infection or involvement of the prosthesis. Nevertheless, following standard infection management protocols, the femoral head and acetabular liner were exchanged, and the wound was closed primarily. Empirical intravenous antibiotic therapy with cefuroxime 1.5 g three times daily was initiated. After four days, due to persistently elevated and secondarily rising CRP levels, clindamycin 600 mg three times daily was added. Intraoperative microbiological cultures obtained during this first revision were negative. Cefuroxime alone, followed by the combination of cefuroxime and clindamycin, was administered for a total of seven days after the first revision.

Second revision and further work-up 

Despite the surgical intervention and antibiotic escalation, the patient again developed erythema, warmth, and secretion with recurrent pustule formation at the wound, accompanied by an increase in CRP up to 223 mg/L. During this period, the patient continued to experience severe, constant wound pain that did not respond adequately to conventional postoperative analgesia and was again disproportionate to the absence of deep infection. Under the ongoing suspicion of wound infection, a second revision was performed. Inflamed cutaneous tissue was excised, and multiple new microbiological samples and a histopathologic specimen were taken. 

Intraoperatively, the inflammatory process was again confined to the skin and subcutaneous fat without involvement of fascia, muscle, or the arthroplasty components. The wound was initially left open and treated with Betaisodona-soaked dressings. Three days later, a negative-pressure wound therapy (vacuum-assisted closure (VAC)) system was applied. 

Microbiologically, *Staphylococcus epidermidis* was isolated in one of four samples only after prolonged incubation and was interpreted as contamination. During VAC therapy, *Cutibacterium acnes* grew in a single culture after extended incubation, without convincing evidence of prosthetic involvement. In view of the inconclusive microbiological results and the superficial distribution of inflammation, the antibiotic regimen was switched to intravenous ampicillin/sulbactam. Ampicillin/sulbactam was continued for a further four days as therapeutic broad-spectrum therapy and was then de-escalated to an oral low-dose prophylactic antibiotic regimen, which was maintained for approximately six weeks before antimicrobial therapy was completely discontinued once PG was confirmed, and deep infection had been excluded.

Given the atypical clinical course, negative or inconsistent cultures, and restriction of inflammation to cutaneous and subcutaneous tissues, a dermatology consultation was requested. 

Final diagnosis and treatment 

**Figure 3:**
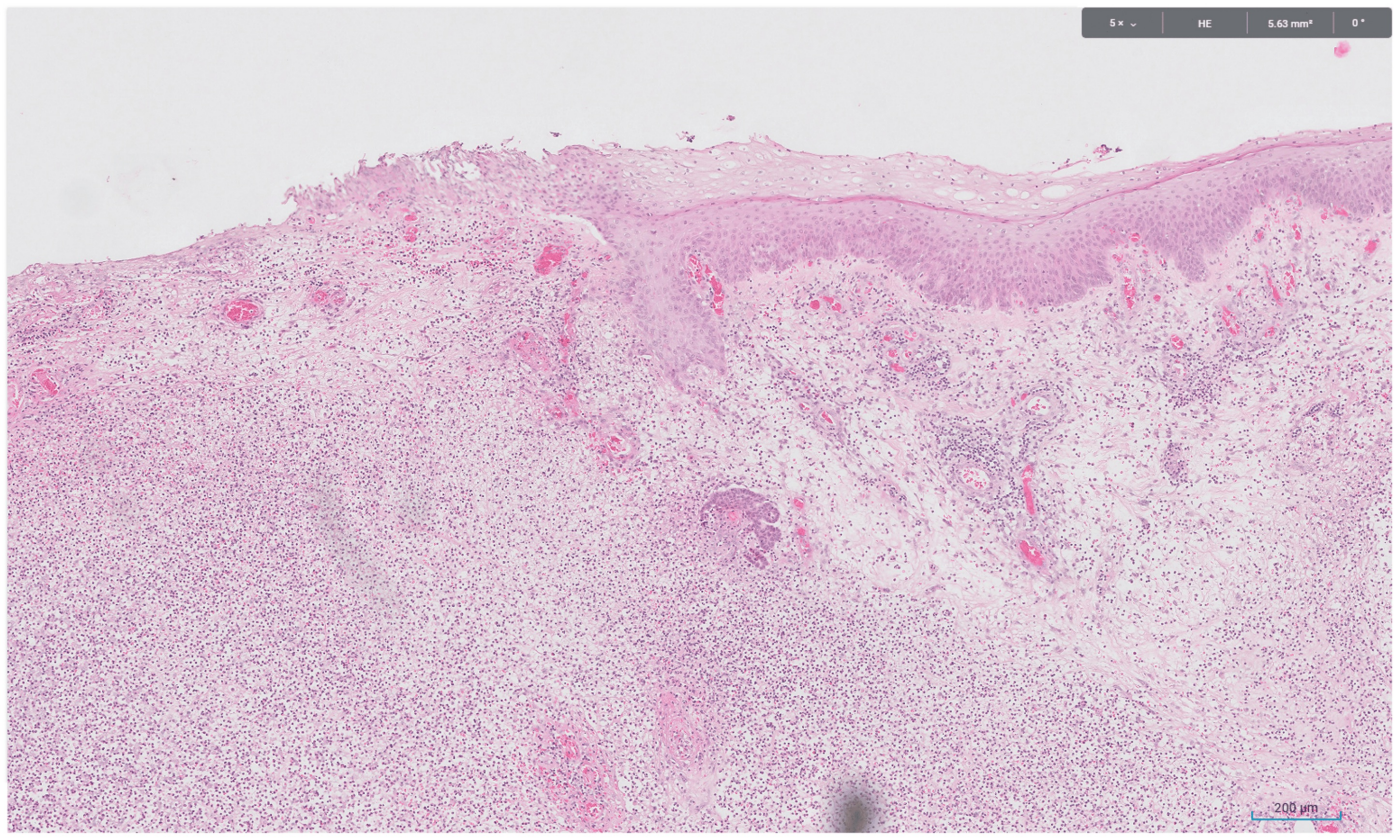
Histopathologic examination (hematoxylin and eosin staining) of a biopsy specimen from the wound edge showing a dense neutrophilic infiltrate with dermal edema and focal necrosis.

Dermatologic evaluation raised a strong suspicion of PG based on the rapid progression of painful pustulo-ulcerative lesions, violaceous, undermined borders, and the history of surgical trauma at the site. Histopathologic analysis of the biopsy specimen revealed a dense neutrophilic infiltrate in the dermis with marked edema and focal necrosis, without evidence of vasculitis, granulomatous inflammation, or significant fibrinoid necrosis of vessel walls. There was no accumulation of microorganisms on special stains, and no histologic features suggestive of leukocytoclastic vasculitis, necrotizing fasciitis, or cutaneous vasculopathy. These findings were consistent with a sterile neutrophilic dermatosis and supported the diagnosis of PG (Figure 3). Taken together with the rapidly progressive, disproportionately painful pustulo-ulcerative lesions, superficial tissue involvement, negative microbiological results, and subsequent improvement under systemic corticosteroid therapy, these findings fulfilled established diagnostic criteria for PG.

In light of this diagnosis, the VAC therapy was discontinued to prevent further mechanical trauma and potential exacerbation of the pathergy phenomenon. Topical wound care and open wound management were continued. Because of the presence of an implanted prosthesis and an open wound, a low-dose prophylactic antibiotic regimen was maintained.

A systemic corticosteroid regimen using oral prednisolone after the appearance of early granulation tissue was employed, starting with a moderate-dose schedule and followed by a gradual taper over several weeks. Corticosteroids were reduced stepwise once a clear clinical response had been achieved, with continued tapering until sustained remission of PG was documented. Clinically, wound pain and erythema improved within a few days after initiation of systemic corticosteroids, and progressive granulation and secondary wound healing were observed over the subsequent weeks, without evidence of recurrence.

In parallel, the previously severe wound pain gradually subsided over the following days after discontinuation of VAC therapy and initiation of systemic corticosteroids, paralleling the improvement in the local inflammatory findings. 

**Figure 4:**
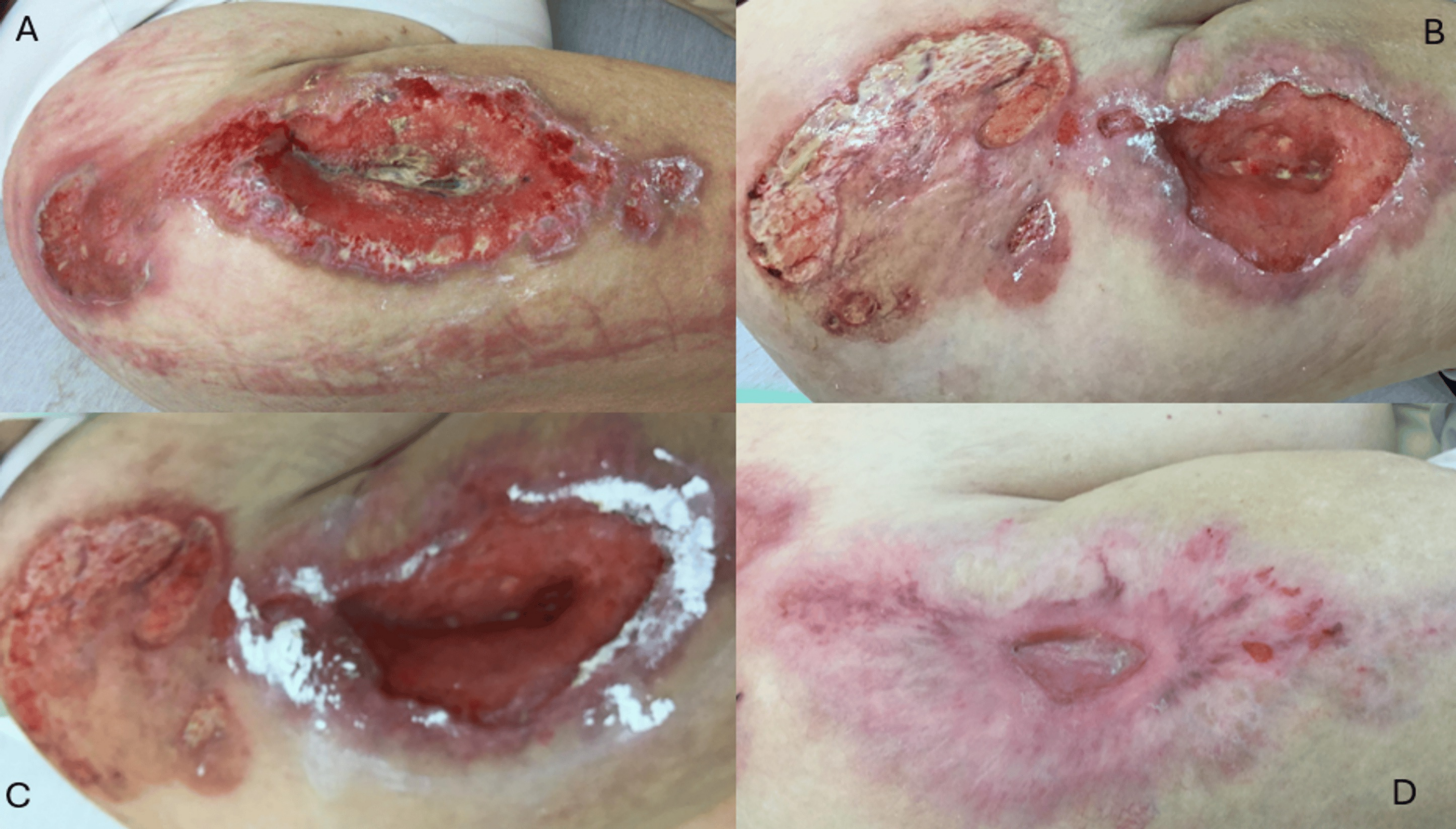
Clinical photographs demonstrating the evolution of the wound. A) Wound appearance three weeks after the second debridement, showing extensive tissue defect with violaceous undermined borders. (B) Wound condition seven weeks after the second debridement and initiation of systemic corticosteroid therapy, demonstrating progressive granulation tissue formation. (C) Advanced healing stage three months after discontinuation of vacuum-assisted closure therapy with open wound management and continued corticosteroid treatment. (D) Final wound appearance at seven weeks showing secondary wound closure and complete epithelialization.

Under this combined approach of topical wound care and systemic corticosteroids, the local wound condition progressively improved (Figure 4). The ulcerations slowly regressed, and secondary wound healing was achieved without evidence of deep infection or need for prosthesis removal. 

In summary, the patient received 11 days of therapeutic broad-spectrum antibiotics followed by approximately six weeks of prophylactic antibiotic therapy before all antimicrobials were stopped.

**Figure 5:**
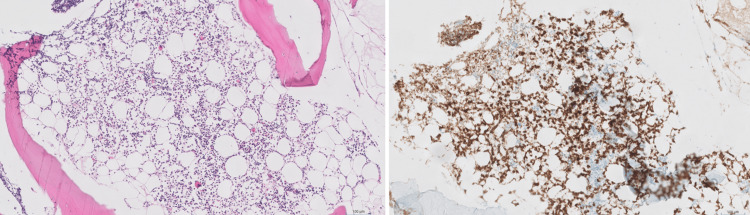
Histological images of bone marrow infiltration in hairy cell leukemia (left: hematoxylin and eosin stain, 100×; right: immunohistochemical CD20 stain, 100×).

Given the recognized association between PG and hematologic disease, further hematologic work-up was undertaken. Bone marrow biopsy revealed a hypercellular marrow with interstitial infiltration by small to medium-sized lymphoid cells with abundant pale cytoplasm and irregular, “hairy” cytoplasmic projections. Immunohistochemical staining demonstrated strong expression of CD20 in the infiltrating cells (Figure 5), findings that were consistent with a mature B-cell neoplasm and supported the diagnosis of hairy cell leukemia as a likely systemic background condition for PG in this patient ​[4]. 

Given the patient’s advanced age and comorbidities, the hematology team initially opted for conservative management with close monitoring rather than immediate hairy cell leukemia-directed systemic therapy during the acute postoperative course, and there was no evidence that specific anti-leukemic treatment influenced the early evolution of PG in this case.

## Discussion

PG is an uncommon but serious complication in the postoperative setting and can be particularly challenging to diagnose after orthopaedic procedures such as total hip arthroplasty [4-6]. In a systematic review of PG after orthopedic or traumatologic surgery, Ebrad et al. identified 31 cases, most involving the lower limb, with symptom onset between two and 17 days postoperatively and a high rate of initial misdiagnosis as infection ​[5]. Postsurgical PG typically presents with severe pain, rapidly progressive erythema, pustules, and ulceration at or near the surgical incision, often with purulent-appearing exudate and elevated inflammatory markers ​[3,5,9]. 

Several diagnostic criteria for PG have been proposed, typically combining a major criterion of a compatible histopathologic neutrophilic infiltrate with minor criteria such as rapid progression of a painful ulcer, exclusion of infection, pathergy, associated systemic disease, and response to immunosuppressive therapy. In our patient, the diagnosis of postoperative PG was supported by a dense neutrophilic infiltrate on histopathology, rapid development of painful pustulo-ulcerative lesions at the surgical site, superficial distribution of inflammation with negative or inconclusive cultures, clinical deterioration after repeated debridements and VAC therapy consistent with pathergy, associated hairy cell leukemia, and a favourable response to systemic corticosteroids [10].

Several case reports have described PG after hip hemiarthroplasty or total hip arthroplasty, with clinical courses similar to that of our patient ​[6-8]. Laskaratou et al. reported an 86-year-old woman who developed PG after hip hemiarthroplasty; she underwent revision surgery and was treated for presumed infection before dermatologic assessment and biopsy confirmed PG ​[7]. Mizushima et al. described a 69-year-old woman with myelodysplastic syndrome who developed PG approximately one week after total hip arthroplasty, initially managed as an infection before PG was recognized and treated with systemic corticosteroids ​[6]. She et al. reported a patient with a femoral neck fracture and known PG who underwent total hip arthroplasty; in that case, careful perioperative immunomodulation was required to prevent disease exacerbation ​[8]. 

Our case shares many of the features reported in these series and case reports. The patient was an elderly woman who developed postoperative erythema, pustules, and purulent-hemorrhagic secretion at the surgical site around postoperative day 6-7. Two revision procedures with extensive debridement and head/liner exchange were performed under the presumption of early periprosthetic joint infection, and broad-spectrum antibiotics were administered, despite the fact that inflammation was confined to skin and subcutaneous tissue and cultures were negative or showed only contaminants. This pattern is consistent with the literature, where delayed diagnosis of PG often results in multiple unnecessary surgical revisions ​[5-7,9]. 

A central mechanism in postsurgical PG is the pathergy phenomenon, whereby minor trauma such as incisions, debridement, or negative-pressure wound therapy can trigger or exacerbate lesions ​[3,9]. The practical review by Guliyeva et al. emphasized that aggressive surgical management and VAC therapy may worsen PG and that once PG is suspected, surgical manipulation should be minimized and immunosuppressive therapy prioritized ​[9]. Similarly, a retrospective study on the surgical approach to PG suggested that limited, carefully timed debridement in combination with systemic therapy is preferable to repeated extensive surgery ​[11]. In our patient, repeated surgical manipulation and VAC therapy likely contributed to the progression of the lesions before diagnosis; after discontinuation of VAC and initiation of corticosteroids, the wound gradually improved and healed secondarily. Recognition of this pathergy pattern in the setting of persistent wound deterioration despite repeated debridement and VAC therapy may facilitate earlier identification of postoperative PG and help avoid unnecessary surgical interventions.

Histopathologically, the dense dermal neutrophilic infiltrate with edema and necrosis in the absence of vasculitis, granulomas, or microorganisms helped distinguish PG from leukocytoclastic vasculitis, necrotizing soft-tissue infection, and other causes of postoperative ulceration. The association between PG and hematologic malignancies is well documented, and recent analyses indicate that hematologic malignancy-associated PG may be associated with worse overall survival compared with PG associated with other conditions ​[4].

In the orthopaedic context, Mizushima et al. reported PG after total hip arthroplasty in a patient with myelodysplastic syndrome receiving granulocyte colony-stimulating factor ​[6]. In our patient, the diagnosis of PG prompted a systematic hematologic evaluation, during which hairy cell leukemia was detected, extending the spectrum of hematologic neoplasms associated with PG. The presence of chronic mild leukocytopenia with relative lymphocytosis, in combination with PG, highlights the importance of carefully reviewing hematologic parameters in such patients, as subtle abnormalities may provide an early clue to an underlying hematologic malignancy. The complete blood count profile, together with bone marrow findings, supported the diagnosis of hairy cell leukemia. This underscores the importance of carefully reviewing hematologic parameters in patients with postoperative pyoderma gangrenosum, as subtle abnormalities may provide an early clue to an underlying hematologic malignancy. Taken together, these findings underscore the need for a thorough systemic work-up whenever PG is diagnosed.

From a therapeutic standpoint, PG is primarily treated with systemic immunosuppression rather than surgical eradication of infection. Systemic corticosteroids are considered first-line therapy and are often combined with other immunosuppressive or biologic agents in refractory cases ​[3,9]. In postsurgical PG, early involvement of dermatology, prompt biopsy, and rapid initiation of systemic therapy are critical to prevent disease progression, limit tissue loss, and avoid unnecessary surgical procedures ​[5,6,9,11]. For patients with a prosthetic joint, management must carefully balance the need for immunosuppression against the risk of true prosthetic joint infection, making close interdisciplinary collaboration between orthopaedic surgeons, dermatologists, and hematologists essential. 

This case reinforces several important clinical messages. First, in patients with atypical postoperative wound deterioration after hip arthroplasty, characterized by severe pain, violaceous undermined borders, superficial involvement of skin and subcutaneous tissue, negative or inconsistent cultures, and worsening after debridement or VAC, PG should be considered early in the differential diagnosis ​[5,7,9]. Second, early dermatologic assessment and histologic confirmation can prevent repeated unnecessary surgeries and facilitate the timely initiation of appropriate immunosuppressive treatment. Third, the detection of PG should prompt evaluation for associated systemic diseases, including hematologic malignancies, as illustrated by the diagnosis of hairy cell leukemia in our patient ​[4]. 

Clinical message

PG should be considered in patients presenting with rapidly progressive, disproportionately painful ulcerations at the incision site after total hip arthroplasty, particularly when cultures remain negative, and inflammation is confined to skin and subcutaneous tissue. Repeated debridements, component exchanges, and aggressive negative-pressure wound therapy may paradoxically worsen PG due to pathergy and should be avoided once PG is suspected. Early dermatologic consultation, skin biopsy, and timely initiation of systemic corticosteroids or other immunosuppressive agents are essential for optimal outcomes. Importantly, the diagnosis of PG warrants thorough evaluation for associated systemic diseases, especially hematologic malignancies such as hairy cell leukemia, which may significantly impact both prognosis and overall management strategy.

## Conclusions

Postoperative PG following elective total hip arthroplasty is a rare but serious condition that closely mimics early periprosthetic joint infection and is frequently misdiagnosed. Early onset of painful pustulo-ulcerative lesions at the incision site, superficial distribution of inflammation, negative cultures, and exacerbation after repeated debridement or negative-pressure wound therapy should raise suspicion of PG. Early dermatologic consultation, histopathologic confirmation, and initiation of systemic immunosuppressive therapy are crucial to prevent further tissue damage and unnecessary surgical procedures. Recognition of the strong association between PG and hematologic malignancies underscores the importance of a comprehensive systemic evaluation when PG is diagnosed. 

## References

[1] Monari P, Moro R, Motolese A (2018). Epidemiology of pyoderma gangrenosum: results from an Italian prospective multicentre study. Int Wound J.

[2] George C, Deroide F, Rustin M (2019). Pyoderma gangrenosum - a guide to diagnosis and management. Clin Med (Lond).

[3] Domingue G, Cox K, Fox JA, Atkins A, Haleem AM, Brewer J (2023). Spontaneous diffuse pyoderma gangrenosum after polytrauma and orthopedic fixation: a case report with brief review of literature. J Orthop Case Rep.

[4] Kridin K, Ankary-Khaner M, Kridin M, Cohen AD, Badarny S (2024). Hematological malignancy-associated pyoderma gangrenosum: evaluating the magnitude of the association. Front Med (Lausanne).

[5] Ebrad S, Severyns M, Benzakour A, Roze B, Derancourt C, Odri GA, Rouvillain JL (2018). Pyoderma gangrenosum after orthopaedic or traumatologic surgery: a systematic revue of the literature. Int Orthop.

[6] Mizushima M, Miyoshi H, Yonemori K (2021). Pyoderma gangrenosum after total hip arthroplasty associated with administration of granulocyte colony-stimulating factor: a case report. JBJS Case Connect.

[7] Laskaratou E, Trygonis N, Dimitriou R, Kouvidis G (2022). Pyoderma gangrenosum after hip hemiarthroplasty; a case report. Trauma Case Rep.

[8] She GR, Chen JY, Zhou ZQ, Zha ZG, Liu N (2017). Total hip arthroplasty for femoral neck fracture with pyoderma gangrenosum patient: a case report. Int J Surg Case Rep.

[9] Guliyeva G, Janis JE (2024). Postsurgical pyoderma gangrenosum requiring plastic surgical intervention: a practical review. Plast Reconstr Surg Glob Open.

[10] Kaushik RD, Ansari A, Sharma D, Ahlawat R, Sharma N (2026). C-reactive protein and hypertension grade in newly detected, treatment naïve adults: a case-control study. Int J Drug Deliv Technol.

[11] Bingoel AS, Krezdorn N, Kaltenborn A, Dastagir K, Jokuszies A, Mett TR, Vogt PM (2021). The surgical approach to pyoderma gangrenosum: a retrospective monocenter study. Wound Repair Regen.

